# Facial length and angle feature recognition for digital libraries

**DOI:** 10.1371/journal.pone.0306250

**Published:** 2024-07-24

**Authors:** Shuangyan Li, Min Ji, Ming Chen, Lanzhi Chen

**Affiliations:** 1 School of Art and Design, Qingdao University of Technology, Qingdao, China; 2 Department of Fine Arts, Cangzhou Normal University, Cangzhou, China; 3 College of Arts and Sports, Dankook University, Yongin, South Korea; International University of Languages and Media: Libera Universita di Lingue e Comunicazione, ITALY

## Abstract

With the continuous progress of technology, facial recognition technology is widely used in various scenarios as a mature biometric technology. However, the accuracy of facial feature recognition has become a major challenge. This study proposes a face length feature and angle feature recognition method for digital libraries, targeting the recognition of different facial features. Firstly, an in-depth study is conducted on the architecture of facial action networks based on attention mechanisms to provide more accurate and comprehensive facial features. Secondly, a network architecture based on length and angle features of facial expressions, the expression recognition network is explored to improve the recognition rate of different expressions. Finally, an end-to-end network framework based on attention mechanism for facial feature points is constructed to improve the accuracy and stability of facial feature recognition network. To verify the effectiveness of the proposed method, experiments were conducted using the facial expression dataset FER-2013. The experimental results showed that the average recognition rate for the seven common expressions was 97.28% to 99.97%. The highest recognition rate for happiness and surprise was 99.97%, while the relatively low recognition rate for anger, fear, and neutrality was 97.18%. The data has verified that the research method can effectively recognize and distinguish different facial expressions, with high accuracy and robustness. The recognition method based on attention mechanism for facial feature points has effectively optimized the recognition process of facial length and angle features, significantly improving the stability of facial expression recognition, especially in complex environments, providing reliable technical support for digital libraries and other fields. This study aims to promote the development of facial recognition technology in digital libraries, improve the service quality and user experience of digital libraries.

## 1. Introduction

With the widespread application and development of digital libraries, the role of facial recognition is becoming increasingly important [1]. The application of facial length and angle feature recognition technology can improve the intelligent and personalized service quality of libraries [2]. On a global scale, the construction of digital libraries is no longer just a repository of knowledge, but also a creator and distributor of knowledge, becoming the center of communities and an important place for learning [3]. The recognition technology of facial expression diversity is widely used in various industries and fields, and this technology plays a very important role in enhancing user interaction experience [4]. However, there are countless studies on FL-AF recognition technology, but there is relatively little research on the digital library environment [5]. Although existing facial recognition technologies have been applied in various scenarios, they still face challenges in accurately capturing and recognizing facial length and angle features, especially in specific environments such as digital libraries. In response to this challenge, research is proposed on facial length and angle feature recognition for digital libraries.

The innovation of the research lies in two aspects. Firstly, to address the difficulty of complex feature facial recognition, a recognition technology for multiple facial features is proposed, providing reference for complex scene facial recognition. Secondly, it is important to consider human facial expressions and further optimize recognition techniques to enhance the reliability of recognition techniques.

The contribution of research is reflected in two aspects, one of which is that the research content will provide technical support for the construction of digital libraries. The second is to further improve the shortcomings of facial recognition technology and enhance its security.

**Fig 1:**
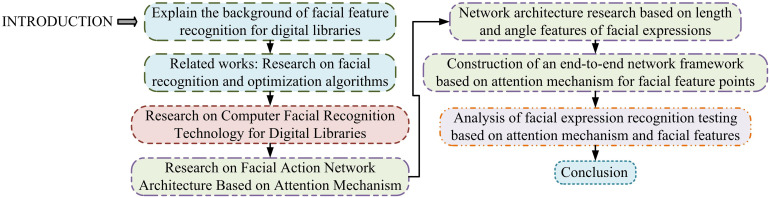
Research process.

The research content is mainly divided into four section. The first part is a literature review, which comprehensively reviews the application of facial length and angle feature recognition in digital libraries, as well as the current research status of various scholars on their recognition technologies. The second part is to study recognition algorithms based on deep learning. The first section outlines the principles of deep learning in facial length and angle feature recognition. The second section studies and optimizes the recognition of facial length and angle features. The third section constructs a facial length and angle feature recognition model based on an improved recognition algorithm. The third part comprehensively tests the performance of deep learning based facial length feature and angle feature recognition. The fourth part is a summary and outlook. The research process diagram is shown in Fig 1.

## 2. Related works

With the development and popularization of digital libraries, the implementation of facial feature recognition has attracted widespread attention from many scholars, among which significant progress has been made in accuracy and reliability. Y Liu et al. proposed an emotion rich feature learning network based on fragment perception. This network used a segment-based feature encoder with two-level self attention and local global relationship learning to design an emotion intensity activation network to generate emotion activation maps for expression classification. Compared to the most advanced methods currently available, CEFLNet has improved its performance in facial expression recognition (FER) [6]. N B Kar et al. proposed a hybrid feature descriptor and improved classifier combination scheme to solve the FER problem in the field of computer vision. This mixed feature descriptor combines spatial and frequency domain features and had good robustness to lighting and noise. This scheme outperformed existing methods in terms of facial expressions of Japanese women and FER on the extended Cohn Kanade (CK+) dataset [7]. L Zhou et al. proposed a feature refinement method for micro expression recognition, which utilizes specific expression features for learning and fusion to extract salient and discriminative features of micro expressions. It obtained expression-shared features through an optical flow-based initialization module, extracted salient and discriminative features of specific expressions, and predicted category labels by fusing the features of specific expressions. This method has shown effectiveness under different protocols [8]. A Sha proposed a variant method that combines deep neural network models with gravity search algorithms. It first used local binary patterns to extract initial features, and then optimized these features using standard, binary, and fast discrete gravity search algorithms. This method surpassed the current state-of-the-art technology in terms of average recognition accuracy [9]. Y Liu proposed a multi factor joint normalization network based on generative adversarial networks to normalize faces, including complex facial changes such as posture, lighting, and expressions. The introduced identity perception loss enabled the generated facial images to maintain consistent identity features. This method could effectively improve face recognition performance under unconstrained conditions while maintaining identity features [10].

The research in the field of facial recognition mainly focuses on the application of deep learning. The improvement of FER technology through deep learning methods has been widely studied by many scholars to improve the accuracy and efficiency. M Gao et al. proposed a knot defect recognition model that combines convolutional neural networks, attention mechanism (AM), and transfer learning. This model combined the SE module with Basicblock to learn and enhance useful features for the current task, while suppressing useless features. The accuracy of this model in the test set was 98.85%, providing a new approach for non-destructive testing of wood [11]. S Yang et al. proposed a dynamic domain adaptation method based on deep multi autoencoder (DMA) AM. This method first utilized pre trained DMA with 6 different activation functions to construct a DMA network with AM for feature extraction, and then automatically assigned weights to the edges and conditional distributions of learning domain invariant fault features. This method had better superiority and stability, effectively improving the performance of rotating machinery fault diagnosis [12]. M Zhu et al. proposed an intelligent model using transfer learning with AM to simulate and predict dynamic gas adsorption. This model captured the flow details near the breakthrough zone for the first time, and was used to process heterogeneous data from different materials and operating conditions. The model had excellent predictive ability for dynamic ammonia adsorption modeling on MCM-41 matrix materials, with adsorption results reaching 7.032 × 10–8 and 4.609 × 10–8, respectively, proving the effectiveness and superiority of the model [13]. D Niu et al. proposed a new method for short-term multi energy load forecasting based on the CNN-BiGRU model. This method introduced three AM modules into the hidden state of BiGRU, extracted the multi energy coupling relationship using hard weight sharing, and implemented optimization using a new multi task loss function weight optimization method. Compared with traditional LSTM models, this model has improved the accuracy of cold, hot, and electricity load prediction by 61.86%, 73.03%, and 63.39%, respectively [14]. T Hui et al. proposed a universal and more focused crack detection model for aircraft engine blades based on Yolov4 tiny. This model introduced an improved attention module and proposed an optimized non-maximum suppression method, which improved the effectiveness of multi-scale feature fusion. This model exhibited good robustness on different lighting and noise images, with an average accuracy of 81.6% on the integrated dataset, which was 12.3% higher than the original Yolov4 tiny [15].

In summary, existing facial recognition technologies still have poor performance in facial feature recognition under complex environments and variable lighting conditions, with high computational complexity and cost. In order to further improve the user experience and service efficiency of digital libraries, research focuses on optimizing recognition technology based on facial length and angle features, aiming to reduce facial recognition errors in complex situations and reduce the waste of computing resources. To provide faster and more accurate personalized services for digital library users, thereby promoting the improvement of library intelligence level.

## 3. Computer FRT for digital libraries

AM has been introduced in computer FRT research for digital libraries to achieve more accurate capture and processing of facial motion related information. AM can automatically recognize and focus on important features, thereby improving the performance of the model. In the research of FL-AF network architecture for facial expressions, it attempts to identify key length and angle features that affect facial expressions to achieve more accurate facial recognition. In the construction of an end-to-end network framework based on AM facial feature points (FFP), precise positioning of FFP can further improve the accuracy and efficiency of facial recognition. This study aims to provide new evidence for digital libraries and a new perspective for computer FRT.

### 3.1. Facial action network architecture based on AM

**Fig 2:**
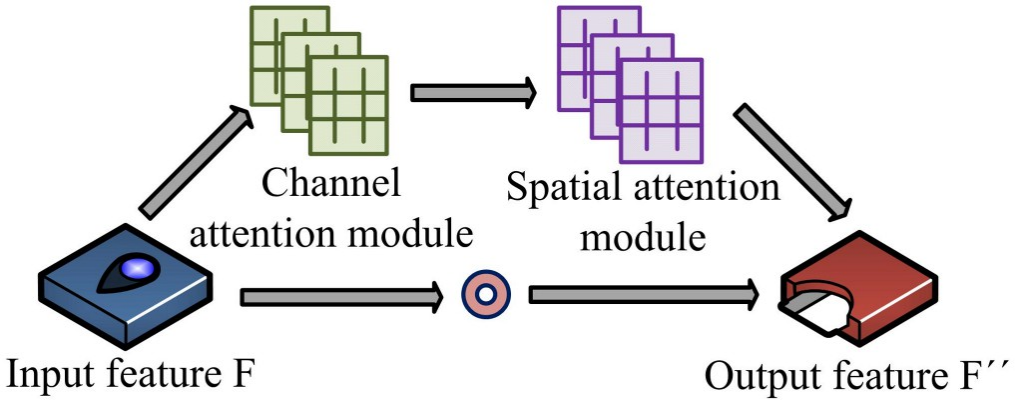
Network structure of attention mechanism integrating channel and space.

In the context of digital libraries, computer FRT can improve the intelligence level of library services and provide convenient and personalized services for readers [16]. However, due to the complex factors involved in facial recognition, such as facial movements, facial expression changes, lighting conditions, etc., FRT poses certain challenges [17, 18]. AM is a mechanism that can automatically recognize and focus on important features. This study introduces AM into the facial action network architecture to capture and process facial action related information [19]. Fig 2 shows the AM network structure that integrates channels and space.

**Fig 3:**
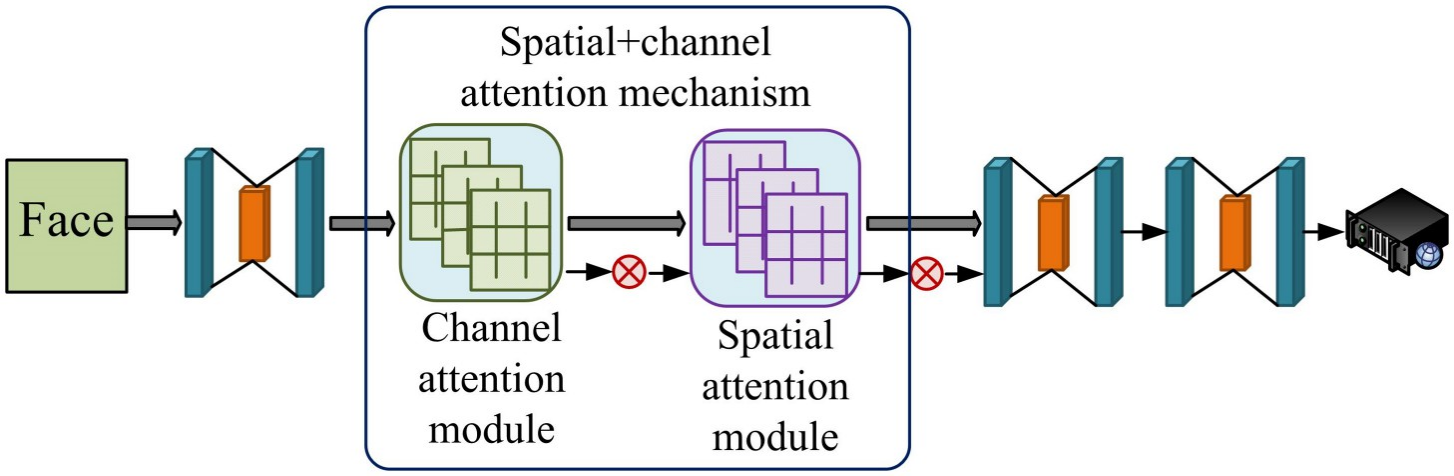
Facial expression recognition network structure incorporating attention mechanism.

The AM structure in Fig 1 is a fusion of AM in both spatial and channel dimensions to capture and learn the features of input data [20]. In the spatial dimension, AM can automatically identify and centrally process important areas in input data, ignoring irrelevant or irrelevant information. In the channel dimension, AM can automatically recognize and focus on important features in the input data. Facial expression recognition network structure incorporating attention mechanism is shown in Fig 3.

The structure of Fig 3 is a novel deep learning model that introduces AM to automatically recognize and focus on key parts of facial expressions [21, 22]. In addition, AM can also enable the model to dynamically adjust the focus area and degree when processing facial expressions, maintaining good recognition performance in complex environments. The feature map of each channel can serve as a feature detector, and through the attention module, the model can learn the channel features that are of great concern. The corresponding calculation formula is Eq (1).


$$N_{c}(F)=\sigma (W_{1}(W_{0}(F_{avg}^{c}))+(W_{1}(W_{0}(F_{a\;\text{max}}^{c})))$$ (1)

In Eq (1), *W*_0_ and *W*_1_ represent the weights of the ReLU activation function activated in the perception model. *N*_*c*_(*F*) represents the channel weight coefficient. *F* represents the input feature map. The spatial attention module (SAM) takes the feature map *F*′ output by the channel attention module (CAM) as the input feature of the module, allowing the network to focus on the input region of the feature map, as shown in Eq (2).


$$N_{s}(F^{\prime })=\sigma (f^{7\times 7}(\left[F_{avg}^{s};F_{\text{max}}^{s}\right]))$$ (2)

In Eq (2), *F*′ represents the input feature on the channel. *N*_*s*_(*F*′) represents the spatial weight coefficients obtained from the output. The feature *F*′ of the output after CAN reinforcement is Eq (3).


$$F\prime =N_{c}(F)⊗F$$ (3)

In Eq (3), *F* represents the input feature. *N*_*c*_(*F*) represents the channel weight coefficients element by element. And the feature *F*″ after SAM reinforcement is Eq (4).


$$F^{\prime \prime }=N_{s}(F^{\prime })⊗F^{\prime }$$ (4)

In Eq (4), *F*′ represents the input feature. *N*_*s*_(*F*′) represents the spatial weight coefficients element by element. This network architecture captures user expressions and emotions, providing a new optimization path for computer FRT for digital libraries.

### 3.2. Network architecture of FL-AF based on facial expressions

**Fig 4:**
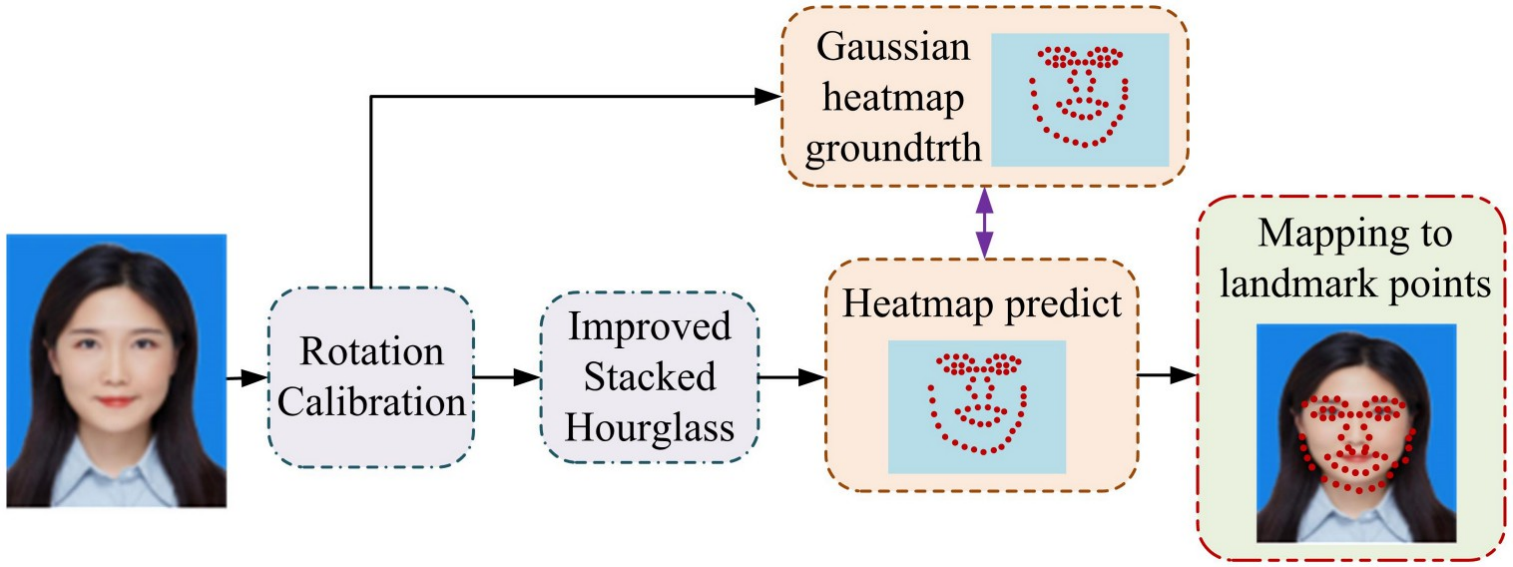
Process of multi angle facial feature point detection algorithm (The character in the Figure is the first author, with her own consent).

The computer FRT for digital libraries has brought significant convenience and improvement to the management and services of libraries, enabling efficient operations such as self-service borrowing, returning, and identity verification [23, 24]. The FL-AF architecture based on facial expressions preprocesses input facial images, including standardization and grayscale. After reducing noise and irrelevant information in the image, to calculate the features of face length and angle [25, 26]. Fig 4 shows the flowchart of the multi angle FFP detection (FA-FFP-D) algorithm. Ethical statement: Strictly adhere to ethical requirements and legal provisions in the research, and obtain review and approval from relevant institutions. The patients (or participants) who participated in the study have already signed an informed consent form. At the same time, we promise to protect the privacy of the research and ensure that the data and information used in the study will not disclose the patient’s personal identity or other sensitive information.

**Fig 5:**
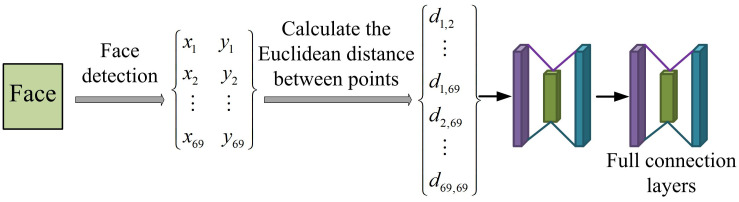
Network structure of facial length features.

The FA-FFP-D algorithm aims to capture and analyze facial feature points from multiple perspectives, as well as process facial images with side faces or non-standard angles [27]. Firstly, it involves image preprocessing, including standardization and grayscale transformation, to remove noise and irrelevant information from the image and capture facial feature points. Furthermore, deep learning models are used to automatically learn and extract image features without the need for pre-defined features. Next is to analyze the image, including frontal angle, lateral angle, and other non-standard angle analysis images. Finally, the feature points from all angles are integrated and trained and optimized using machine learning algorithms. The framework of facial length feature (FLF) is shown in Fig 5.

FLF focuses on capturing and analyzing the length features of faces, such as the distance between eyes, the distance between eyes and mouth, etc. [28, 29]. To learn and extract image features through preprocessing, and use the extracted feature information to calculate the length feature of the face for recognition and classification. Each feature point is denoted as *k*_*i*_, and the coordinates of *k*_*i*_ are (*x*_*i*_, *y*_*i*_). The Euclidean distance between *k*_*i*_ and *k*_*j*_ is defined as *d*_*i*,*j*_. The formula is Eq (5).


$$d_{i,j}=\sqrt{{\left(x_{i}-x_{j}\right)}^{2}+{\left(y_{i}-y_{j}\right)}^{2}}$$ (5)

**Fig 6:**
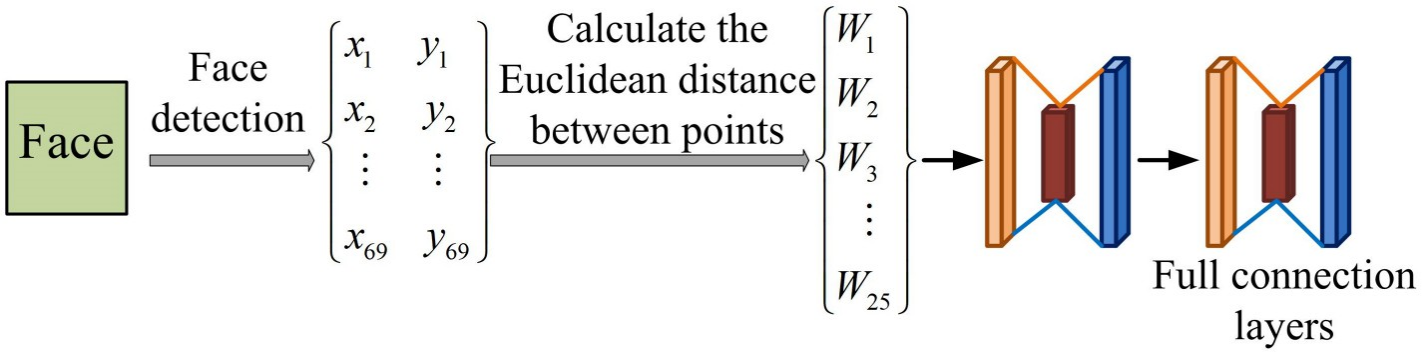
Network structure of facial angle featuresm.

In Eq (5), the value of its length characteristic *D* is (*d*_1,2_*d*_1,3_…*d*_1,68_*d*_2,3_…*d*_2,68_…*d*_67,68_). The structure of facial angle features (FAF) is shown in Fig 6.

FAF is used to capture and analyze angle features of the face, such as the angle between the eyes, nose, and mouth [30]. It is necessary to pre-process the input image, including standardization and grayscale, and then calculate the angle features of the face based on the extracted feature information. The mathematical expression for the angle formed by feature point *k*_1_, *k*_2_, *k*_3_ and feature point *k*_1_, *k*_2_, *k*_3,_ *k*_4_ is Eq (6).


$$\theta =\text{arccos}\frac{\left(x_{1}-x_{2})(x_{3}-x_{2})+(y_{1}-y_{2})(y_{3}-y_{2}\right)}{\sqrt{{\left(y_{1}-y_{2}\right)}^{2}+{\left(y_{1}-y_{2}\right)}^{2}}\cdot \sqrt{{\left(x_{3}-x_{2}\right)}^{2}+{\left(y_{3}-y_{2}\right)}^{2}}}$$ (6)

In Eq (6), the coordinates of feature point *k*_*i*_ are denoted as (*x*_*i*_, *y*_*i*_), *i*∈[1,68]. The length feature *D* = (*d*_1,2_*d*_1,3_…*d*_1,68_*d*_2,3_…*d*_2,68_…*d*_67,68_) and angle feature *W* = (*W*_1_ *W*_2_ … *W*_24_) are extracted and normalized. The commonly used processing methods include Min-Max and Z-Score, where Min-Max normalization specifically refers to the linear transformation that maps the values of the original data to [0,1]. It transforms the sequence *x*_1_, *x*_2_, … *x*_*n*_ as shown in Eq (7).


$$y_{i}=\frac{x_{i}-\text{min}}{\text{max}-\text{min}}(i\neq j)$$ (7)

In Eq (7), min represents the minimum value in sequence *x*_1_, *x*_2_, … *x*_*n*_, and max represents the maximum value. Z-Score normalization refers to the mapping of the mean and standard deviation of the original data, thereby unifying data of different magnitudes. The expression for transforming sequence *x*_1_, *x*_2_, … *x*_*n*_ is Eq (8).


$$y_{i}=\frac{x_{i}-\bar{x}}{s}$$ (8)

In Eq (8), $\bar{x}$ represents the mean in sequence *x*_1_, *x*_2_, … *x*_*n*_, and *s* represents the standard deviation. The computer FRT for digital libraries plays a crucial role in facial expressions and identity recognition, which can improve the service efficiency and user experience of digital libraries.

### 3.3. Construction of an end-to-end network framework based on AM FFP

**Fig 7:**
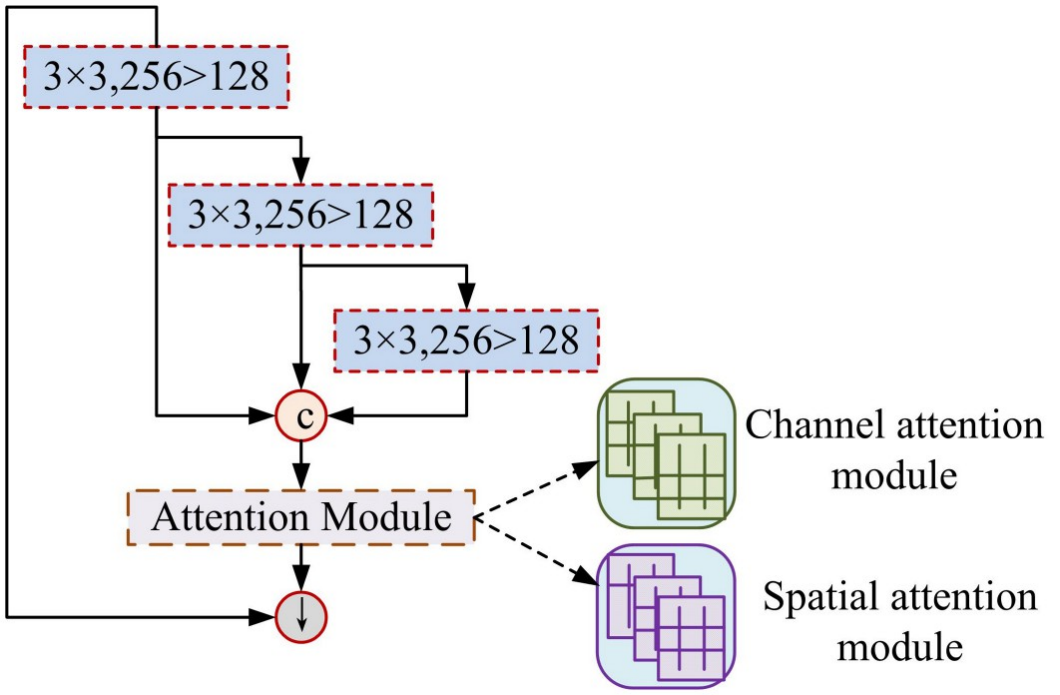
Attention module network.

The computer FRT for digital libraries can achieve unmanned self-service borrowing and returning, provide more personalized reading recommendations, and achieve accurate statistical analysis of library usage [31, 32]. This framework integrates facial recognition and AM technology to enhance the automation management level of digital libraries, thereby improving the service quality of the library. The attention module network is shown in Fig 7.

The network in Fig 7 simulates the AM of the human brain when processing visual information. When processing a large amount of information, the human brain can automatically focus on important parts and ignore unimportant parts [33, 34]. The attention module is also used to weight and filter the features in the input data, focusing more on the key areas of facial features. Decision fusion adds the output results of different classifiers by weight to obtain a target judgment result, and the expression of the decision output result is Eq (9).


$$o_{i}=\alpha p_{i}+(1-\alpha )q_{i}$$ (9)

In Eq (9), *i* ∈ [1, *c*] and *c* represent the number of sentiment classifications. *p*_*i*_, *q*_*i*_ represent the outputs of two network branches. *o*_*i*_ represents the output of the final network. *α* represents the performance of each network, with a range of [0,1]. Among them, the calculation formula for the prediction results of different networks is Eq (10).


$${\tilde{y}}_{3}=\sigma_{3}(l_{1}⊗l_{2})$$ (10)

In Eq (10), *σ*_*s*_ represents the softmax activation function. After obtaining the prediction probabilities ${\tilde{y}}_{1}$, ${\tilde{y}}_{2}$, and ${\tilde{y}}_{3}$ for networks 1, 2, and 3, calculate their respective loss functions as shown in Eq (11).


$$L_{i}=-\sum_{j=1}^{c}y_{i}\;\text{log}({\tilde{y}}_{i,j}),i\in \left\{1,2,3\right\}$$ (11)

In Eq (11), *c* represents the number of types of emoji labels in the dataset. *y*_*j*_ represents the true label value of the *j*-class expression in facial expressions. ${\tilde{y}}_{i,j}$ represents the prediction probability output by network *i*. The overall loss function L is obtained by adding the weights of *L*_1_, *L*_2_, and *L*_3_, and its definition is Eq (12).


$$L=\lambda_{1}L_{1}+\lambda_{2}L_{2}+\lambda_{3}L_{3}$$ (12)

**Fig 8:**
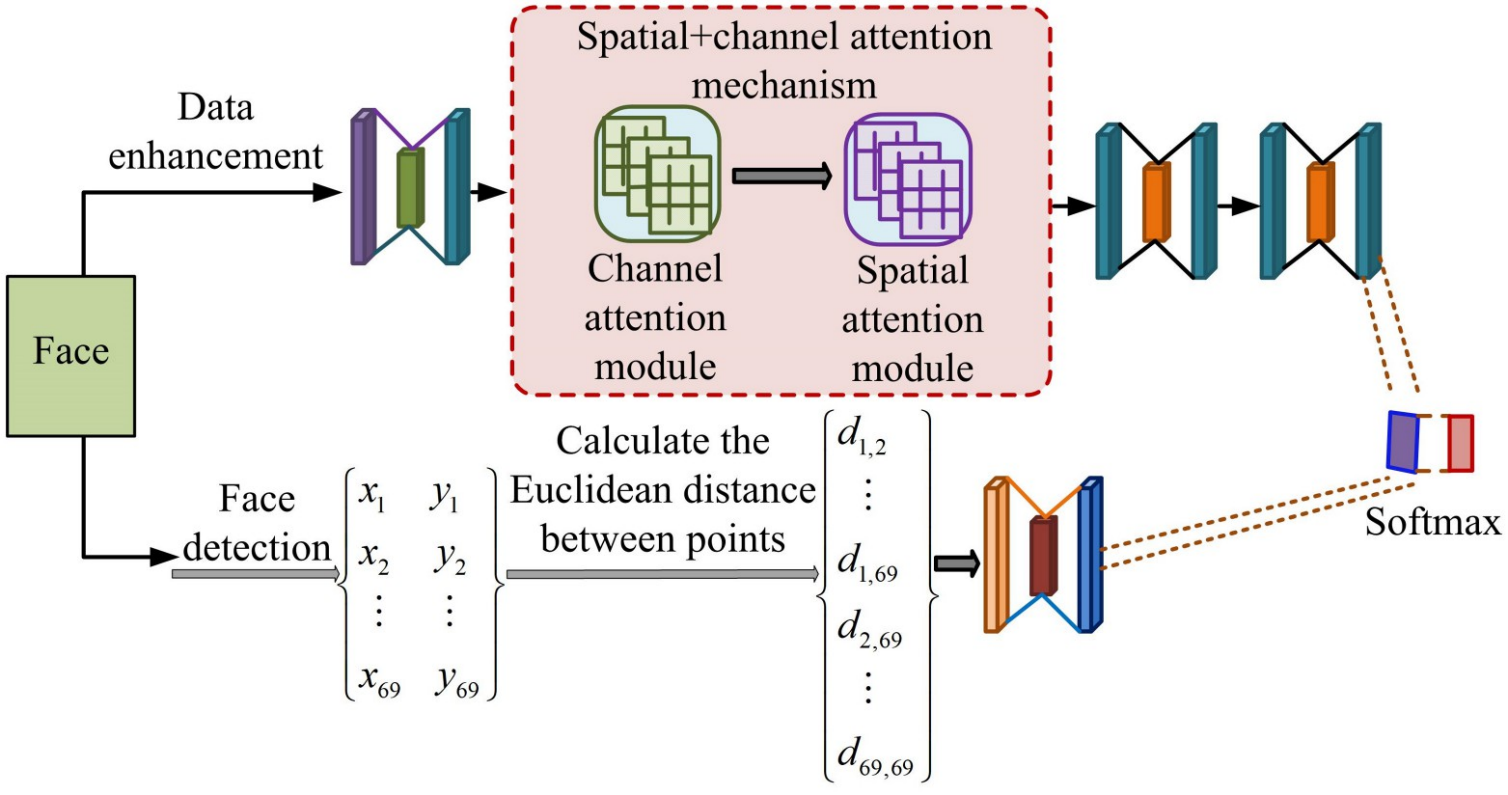
Network structure of facial length and angle features fused with attention mechanism.

In Eq (12), *λ*_1_, *λ*_2_, and *λ*_3_ take values of 1, 1, and 0.1, respectively. This module provides effective tools for handling large-scale data and complex tasks. The structural diagram of FL-AF fused with AM is shown in Fig 8.

The FL-AF structure in Fig 8 is a combination of FLF, FAF, and AM [35]. It preprocesses facial images, automatically learns and extracts image features, calculates their length and angle features, and recognizes facial expressions and identities. The formula for calculating the positioning error between the predicted results of the algorithm and the actual annotated positioning error is Eq (13).


$$e_{a}=\sum_{k=1}^{P}\frac{{\left\|x_{k}-y_{k}\right\|}_{2}}{d}$$ (13)

In Eq (13), *P* represents the number of feature points. *x*_*k*_ and *y*_*k*_ correspond to the true standard and prediction of the *k*-th feature point, respectively. *d* represents the size of the face frame or the distance between the two pupils. The positioning loss function is Eq (14).


$$L_{a}=\frac{1}{N}\sum_{i=1}^{N}\sum_{K=1}^{P}Mse({\hat{H}}_{k}^{o},H_{k}^{g})$$ (14)

In Eq (14), $H_{k}^{g}$ and ${\hat{H}}_{k}^{o}$ represent labeled real heatmaps and predicted heatmaps, respectively. *N* represents the number of training samples. The construction of this network framework achieves the accuracy of facial recognition by automatically focusing and weighting input data to capture facial feature points.

## 4. Analysis of fer testing based on AM facial features

To confirm the accuracy of AM in recognizing facial features, this study configured a unified software and hardware environment to perform FER testing. The experiment used AM and FER-2013 datasets to improve recognition accuracy. In this environment, the facial feature expression recognition test based on AM was run. This environment can provide sufficient computing resources and storage space to ensure the smooth training and testing of the model. Meanwhile, the equipped high-performance graphics card can accelerate the training speed of deep learning models and improve testing efficiency. Table 1 shows the specific experimental parameters.

**Table 1:** Experimental environmental parameter.

Parameter	Specification
Operating System	Windows 10 Professional Edition
Programming Language	Python 3.7
Deep Learning Framework	TensorFlow 2.3
Image Processing Library	OpenCV 4.2.0
Dataset	FER-2013 (Facial Expression Recognition Dataset)
ProcessOI	Intel Core i7-9700K
Memory	32GB DDR4
Graphics Card	NVIDIA GeForce RTX 2080 Ti
Storage	1TB SSD

**Fig 9:**
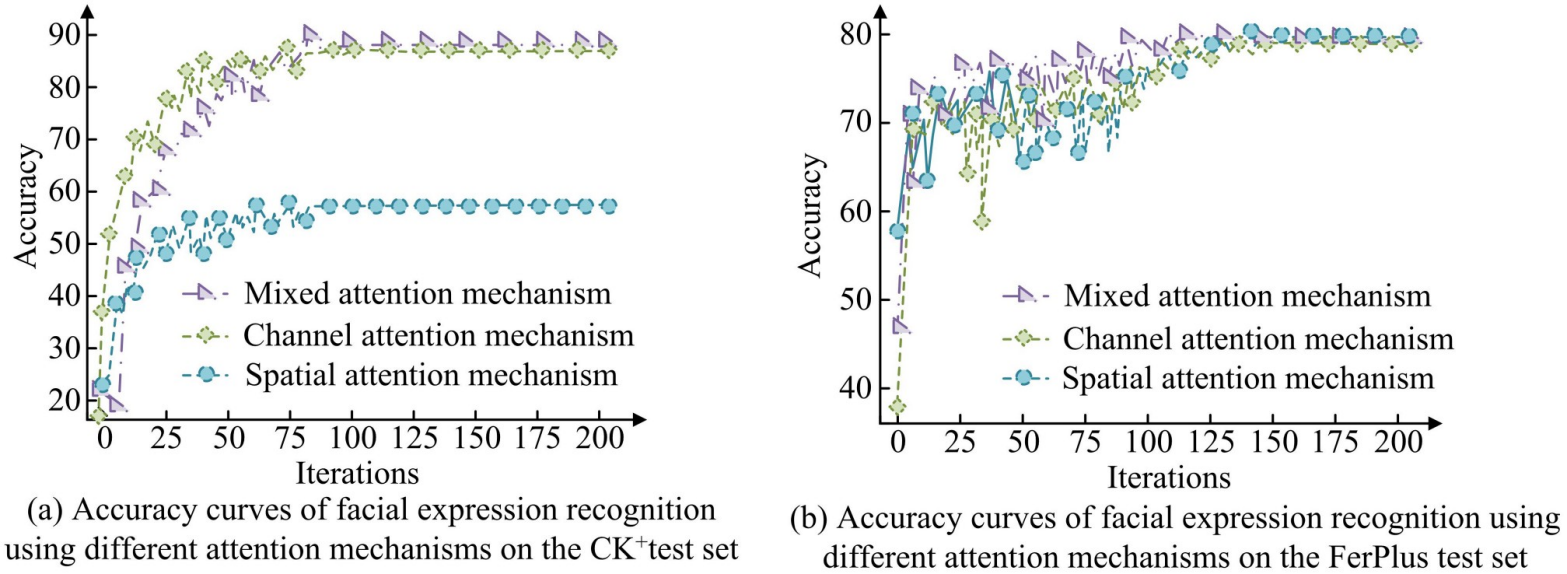
Accuracy results of different attention mechanisms on the CK+and FerPlus test sets.

In Table 1, to ensure the accuracy of the experiment, advanced software tools were used under the Windows 10 Pro operating system, leading FER models were adopted, and unified configuration settings were used in this study. It aims to achieve efficient and accurate expression recognition through this method, providing a basis for further research and application. The accuracy results of different AMs on the CK+and FerPlus test sets are shown in Fig 9.

**Fig 10:**
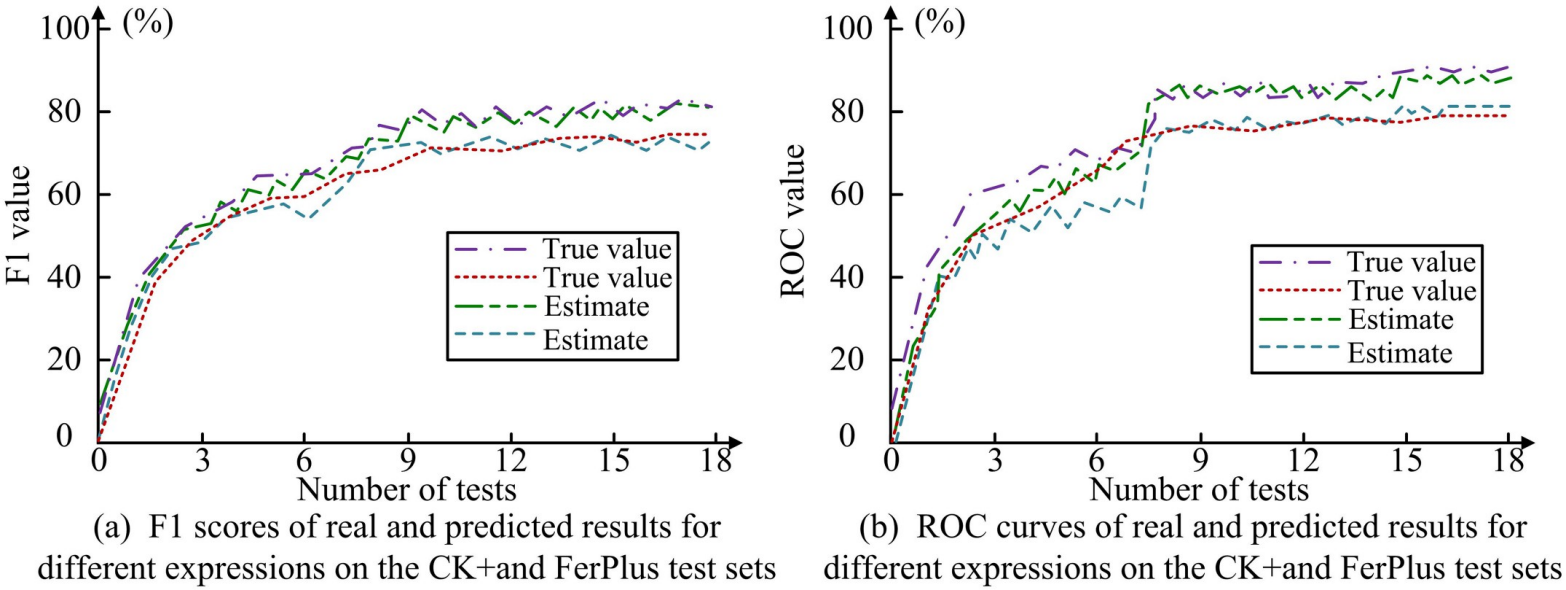
F1 scores and ROC curves of real and predicted results of different expressions on the CK+and FerPlus test sets.

In Fig 9, there is a significant difference in the accuracy of FER using different AMs. In Fig 9(a), the mixed AM performs the best on the CK+test set, with an accuracy of 90.21%. The spatial AM performs the worst with an accuracy of only 58%, highlighting the advantages of hybrid AM. In Fig 9(b), the results on the FerPlus test set also confirm this, with hybrid AM still performing the best with an accuracy of 80.67%, while spatial AM has the lowest accuracy of 79.35%. This once again confirms the importance and effectiveness of hybrid AM in FER. The F1 scores and ROC curve results of the real and predicted results of different expressions on the CK+and FerPlus test sets are shown in Fig 10.

From Fig 10, it can be seen that as the number of tests increases, the true values on the CK+and FerPlus test sets gradually approach the F1 and ROC values of the test values. In Fig 10(a), on the CK+test set, the consistency between the predicted values and the true values reaches its peak at 11 and 9 tests, with accuracy of 80.34% and 76.94%, respectively, indicating that facial expression recognition is the most accurate at this time. In Fig 10(b), on the FerPlus test set, when tested 8 and 9 times respectively, the predicted values had the highest matching degree with the true values, reaching 89.57% and 78.49%, further confirming the high accuracy of facial expression recognition. The effectiveness of the attention mechanism based facial expression recognition model in maintaining high accuracy has been verified, and the model can stably provide high-precision recognition results in multiple tests. Table 2 compares the results of different AMs for different expression recognition rates.

**Table 2:** Different attention mechanisms result in different expression recognition rates.

Test	Expression recognition rate						
Network structure	Happy	Sad	Angry	Amazed	Fear	Neutral	Detest
Spatial attention mechanism	87.51%	85.33%	85.94%	81.86%	87.29%	86.07%	86.91%
Channel attention mechanism	89.36%	87.62%	86.54%	82.94%	88.34%	86.59%	88.73%
Mixed attention mechanism	92.08%	93.46%	95.17%	96.34%	95.41%	93.27%	94.55%

**Fig 11:**
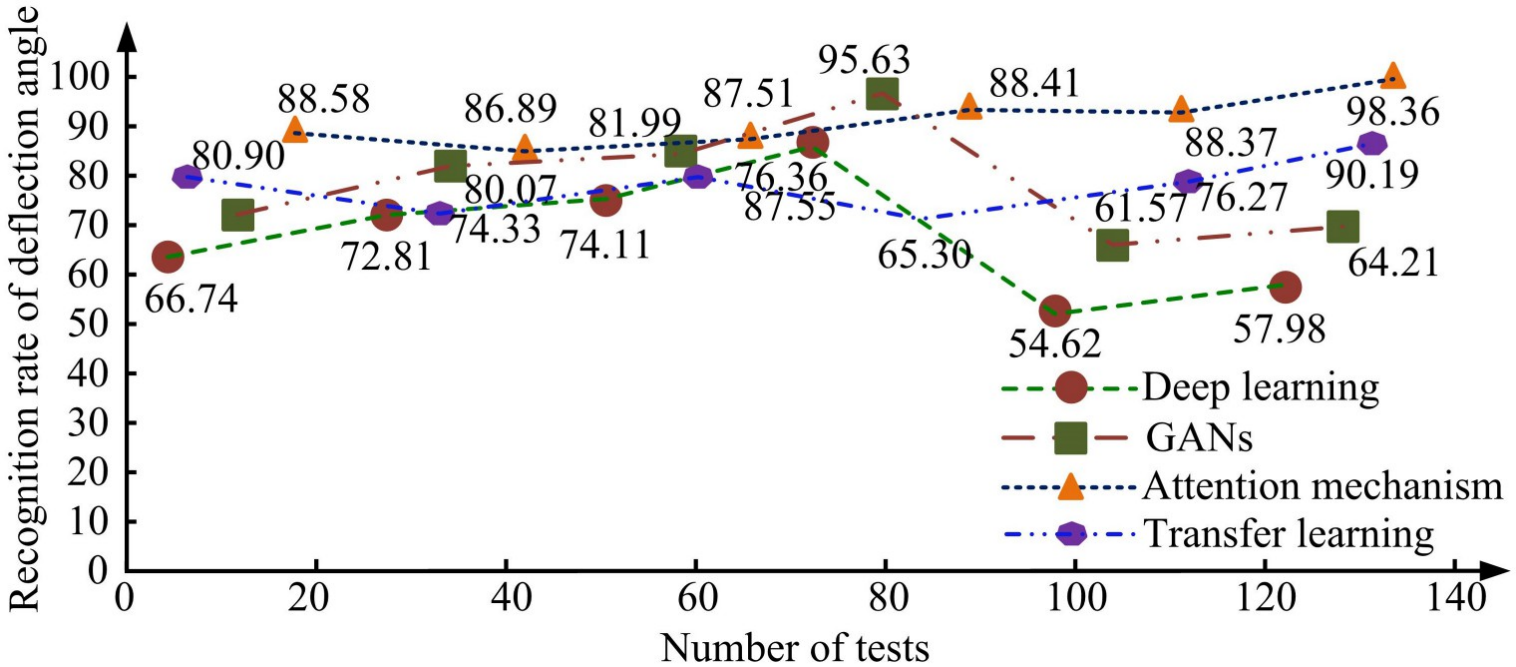
Statistical results of facial expression recognition technology for different angles of human face.

In Table 2, there are significant differences in recognition rates among spatial AM, channel AM, and mixed AM when recognizing expressions of different emotions. The recognition rates of spatial AM for different expressions are 87.51%, 85.33%, 85.94%, 81.86%, 87.29%, 86.07%, and 86.91%, respectively. The recognition rates of channel AM for different expressions were 89.36%, 87.62%, 86.54%, 82.94%, 88.34%, 86.59%, and 88.73%, respectively. The recognition rates of spatial and channel AM for all expressions have significantly improved, reaching 92.08%, 93.46%, 95.17%, 96.34%, 95.41%, 93.27%, and 94.55%, respectively. Therefore, hybrid AM may have better performance in facial expression recognition tasks, improving the accuracy of facial expression recognition. The statistical results of different FER techniques on different angles of facial deviation are shown in Fig 11.

**Fig 12:**
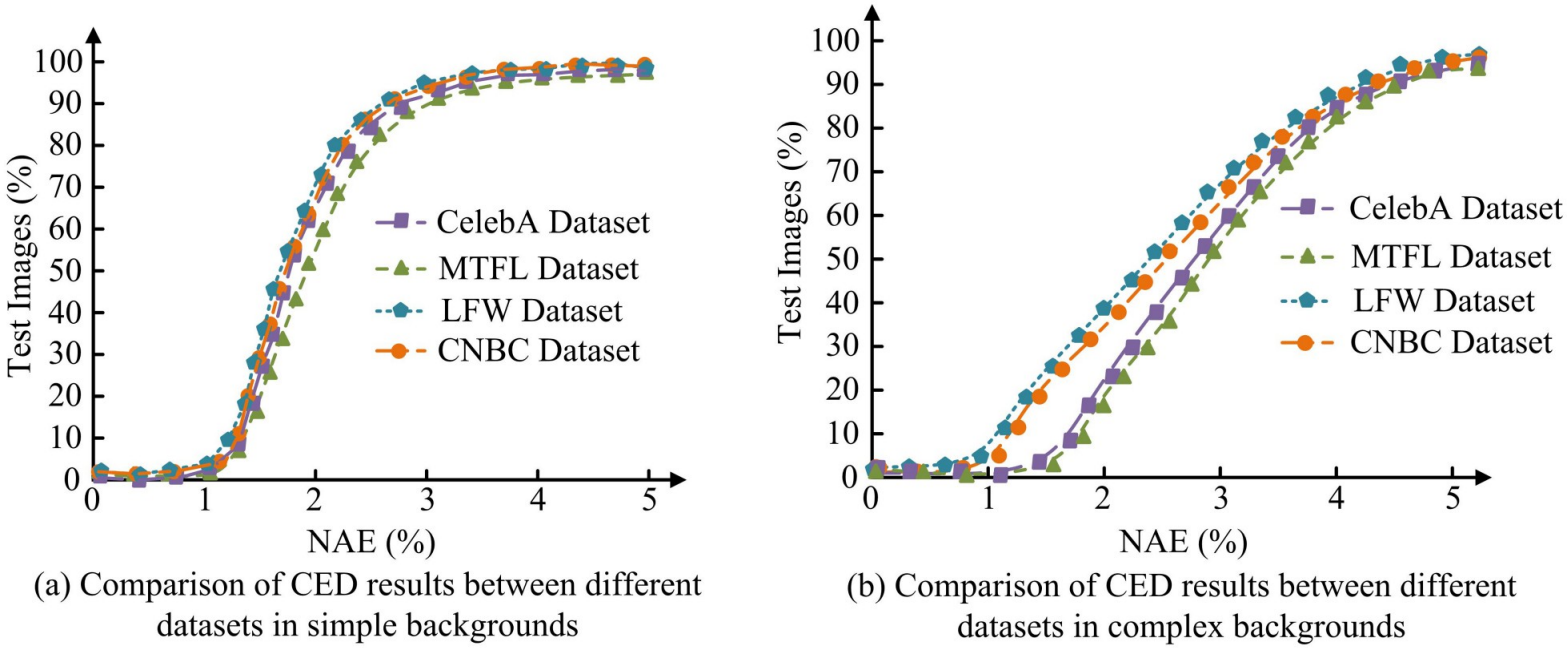
Normalized average error results of fusion attention mechanism facial recognition technology on different datasets.

In Fig 11, there are significant differences among various FER techniques when facing different degrees of facial angle deviation. Among them, as the number of tests increases, the performance of AM and transfer learning technology shows an upward trend, and the recognition rate of AM’s downward deflection angle increases from 88.58 to 98.36. The recognition rate of deflection angle under transfer learning technology increased from 80.90 to 90.19, verifying the effectiveness of transfer learning in FER tasks. The recognition rate of the adversarial generative network (AGN) at the deflection angle decreased from 70.51 to 64.21. The result of angle recognition for deep learning has decreased from 66.74 to 57.98. Therefore, different FER technologies have different performance when facing face angle deviation. The normalized average error (NAE) results of AM FRT fusion on different datasets are shown in Fig 12.

**Fig 13:**
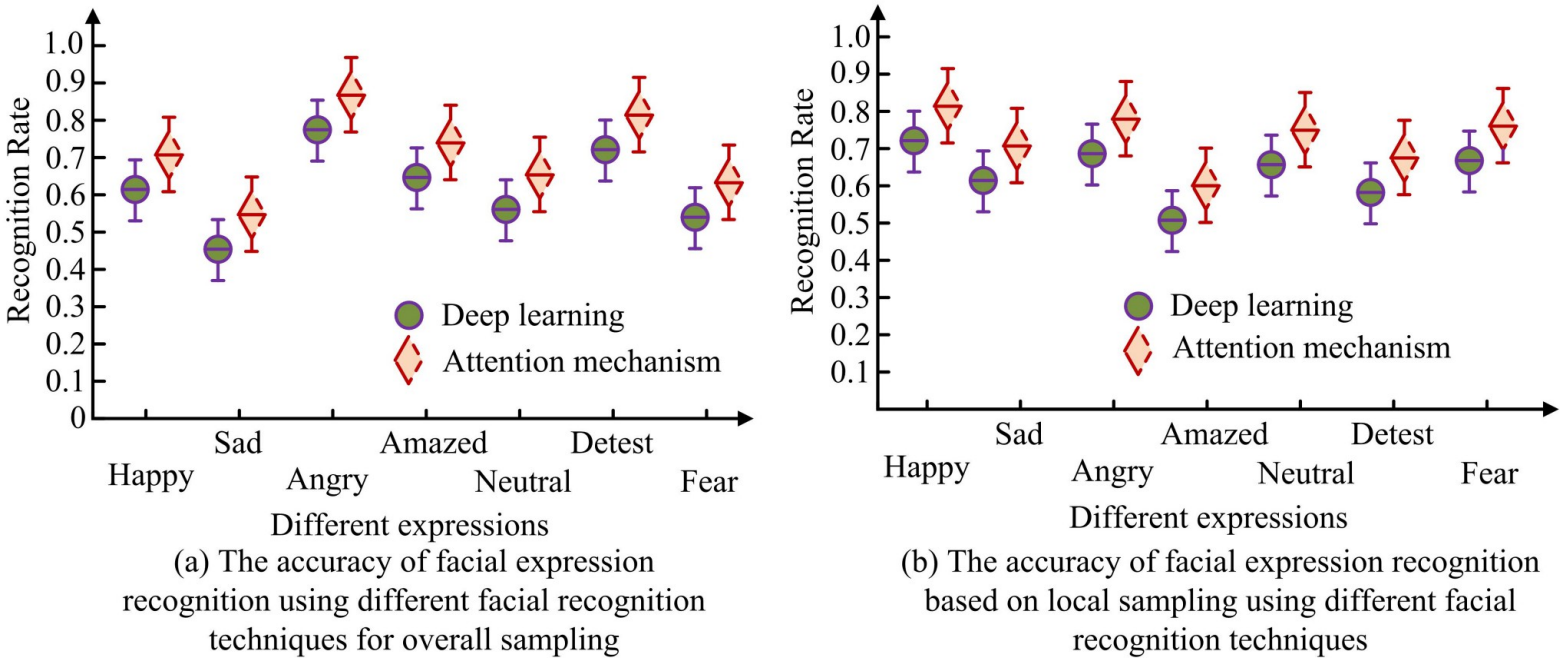
The recognition rate results of attention mechanism and deep learning for different expressions.

In Fig 12(a), the NAE of the CelebA, MTFL, LFW, and CNBC datasets showed a slow upward trend after multiple tests, and tended to stabilize by the fourth test. The NAEs of each dataset are 93.14, 90.35, 97.42, and 95.57, respectively. In Fig 12(b), the NAE of all four datasets showed a rapid upward trend until reaching the final value during the 5th test. The NAE of each dataset is 84.31, 81.69, 89.17, and 86.98, respectively. Therefore, there are significant differences in the NAE performance of different datasets after multiple tests. The recognition rate results of AM and deep learning for different expressions are displayed in Fig 13.

In Fig 13(a), there is a certain difference in the recognition rate of different expressions between AM and deep learning in complex backgrounds. Among them, the recognition rate of sad expressions is the lowest, with recognition rates of 0.54 and 0.48, respectively. However, the recognition rates for angry expressions are relatively high, at 0.89 and 0.82, respectively. Therefore, in complex backgrounds, the characteristics of sad expressions may be more difficult to capture, while anger may be more prominent. In Fig 13(b), the recognition rates of AM and deep learning for different expressions are relatively uniform in a simple background. The recognition rate for surprised expressions is relatively low, with recognition rates of 0.63 and 0.55, respectively. For happy expressions, the recognition rates are relatively highest, at 0.81 and 0.73, respectively. The recognition rates of AM for all expressions are 0.78 and 0.66, respectively. Therefore, AM is more helpful in further improving the accuracy and robustness of FER. Table 3 shows the recognition results of different facial expressions using the fused AM FFP network.

**Table 3:** Different attention mechanisms result in different expression recognition rates.

Expression	Number of tests	Recognition frequency	Average recognition rate
Happy	50	50	99.97%
Sadness	50	48	98.32%
Angry	50	47	97.55%
Amazed	50	50	99.93%
Detest	50	49	98.74%
Fear	50	47	97.18%
Neutral	50	47	97.26%

In Table 3, AM performs FER on FFP, and its recognition rate is generally high. The average recognition rates for happy, sad, angry, surprised, disgusted, fearful, and neutral expressions were 99.97%, 98.32%, 97.55%, 99.93%, 98.74%, 97.18%, and 97.26%, respectively. In 50 tests, expressions of joy and surprise were fully recognized, with recognition rates of 99.97% and 99.93%, respectively. The recognition rates for angry, fearful, and neutral expressions are relatively low, at 97.55%, 97.18%, and 97.26%, respectively, but the recognition frequency is still as high as 47 times. Therefore, although there is a certain gap in expression recognition rate, overall, the expression recognition effect of integrating AM FFP network is still quite outstanding. This verifies that the proposed method can effectively recognize and distinguish different facial expressions, with high accuracy and robustness.

## 5. Conclusion

With the rapid development of artificial intelligence and machine vision technology, the application of FRT in various fields is becoming increasingly widespread. The research on FL-AF recognition for digital libraries has attracted people’s attention. However, due to the complexity of the environment and the diversity of facial features, there are still challenges in improving the accuracy and stability of feature recognition. Therefore, this study proposed a FL-AF recognition study for digital libraries, aiming to evaluate and compare the effects of different AMs in FER, as well as the performance differences of various FER technologies. From this, it can be seen that there are significant differences in the performance of various recognition technologies when facing facial angle deviation. There were significant differences in the performance of AM and deep learning for different expression recognition in complex and simple backgrounds. For example, in complex backgrounds, the recognition rate of sad expressions was the lowest, while the recognition rate of angry expressions was the highest. In summary, this experiment validated the advantages of hybrid AM in FER and compared the performance differences of different FER technologies in handling facial angle deviation. However, there are still limitations to this study, as it did not take into account the more complex actual testing environment and the analysis and comparison of other expressions. Therefore, future research will further explore and optimize FER technology to improve its recognition accuracy and robustness in different backgrounds and angles.

## Supporting information

S1 File Minimal data set definition. (DOCX)
