# Supplementary material for: Facial length and angle feature recognition for digital libraries
Source: PLoS One. 2024 Jul 24;19(7):e0306250. doi: 10.1371/journal.pone.0306250 (PMC11268703; doi:10.1371/journal.pone.0306250)
Supplement: S1 File — (DOCX) [file pone.0306250.s001.docx]

**Minimal Data Set**

The Data in Table 2

| Test | Expression recognition rate | | | | | | |
| --- | --- | --- | --- | --- | --- | --- | --- |
| Network structure | Happy | Sad | Angry | Amazed | Fear | Neutral | Detest |
| Spatial attention mechanism | 87.51% | 85.33% | 85.94% | 81.86% | 87.29% | 86.07% | 86.91% |
| Channel attention mechanism | 89.36% | 87.62% | 86.54% | 82.94% | 88.34% | 86.59% | 88.73% |
| Mixed attention mechanism | 92.08% | 93.46% | 95.17% | 96.34% | 95.41% | 93.27% | 94.55% |

The Data in Table 3

| Expression | Number of tests | Recognition frequency | Average recognition rate |
| --- | --- | --- | --- |
| Happy | 50 | 50 | 99.97% |
| Sadness | 50 | 48 | 98.32% |
| Angry | 50 | 47 | 97.55% |
| Amazed | 50 | 50 | 99.93% |
| Detest | 50 | 49 | 98.74% |
| Fear | 50 | 47 | 97.18% |
| Neutral | 50 | 47 | 97.26% |

The Data in Figure 9

| / | Iterations | 25 | 50 | 75 | 100 | 125 | 150 | 175 | 200 |
| --- | --- | --- | --- | --- | --- | --- | --- | --- | --- |
| CK+test setAccuracy | Mixed attention mechanism | 62.15 | 83.27 | 89.75 | 86.33 | 85.93 | 85.89 | 86.16 | 87.08 |
|  | Channel attention mechanism | 69.89 | 82.14 | 85.47 | 85.61 | 85.58 | 85.91 | 86.04 | 86.16 |
|  | Spatial attention mechanism | 53.06 | 55.18 | 57.31 | 56.17 | 56.09 | 56.08 | 56.11 | 56.09 |
| / | Iterations | 25 | 50 | 75 | 100 | 125 | 150 | 175 | 200 |
| FerPlus test sets Accuracy | Mixed attention mechanism | 73.25 | 77.86 | 78.16 | 75.34 | 75.17 | 76.22 | 76.34 | 76.42 |
|  | Channel attention mechanism | 64.19 | 69.73 | 74.18 | 75.49 | 77.35 | 78.14 | 78.35 | 78.59 |
|  | Spatial attention mechanism | 70.08 | 73.59 | 71.46 | 75.57 | 76.58 | 78.88 | 79.01 | 78.91 |

The Data in Figure 10

| / | Number of tests | 3 | 6 | 9 | 12 | 15 | 18 |
| --- | --- | --- | --- | --- | --- | --- | --- |
| F1 value | True value | 43.26 | 58.14 | 65.27 | 78.38 | 78.49 | 80.05 |
|  | Channel attention mechanism | 43.15 | 57.86 | 65.11 | 78.05 | 78.24 | 79.92 |
| / | Number of tests | 3 | 6 | 9 | 12 | 15 | 18 |
| ROC curves | True value | 44.16 | 59.43 | 81.33 | 81.47 | 81.29 | 81.35 |
|  | Channel attention mechanism | 42.67 | 58.13 | 80.24 | 81.01 | 80.95 | 81.14 |

The Data in Figure 11

| / | Number of tests | 20 | 40 | 60 | 80 | 100 | 120 |
| --- | --- | --- | --- | --- | --- | --- | --- |
| Recognition rate of deflection angle | Deep learning | 66.74 | 72.81 | 74.11 | 87.35 | 54.62 | 57.98 |
|  | GANs | 70.12 | 80.07 | 81.99 | 95.63 | 61.57 | 64.21 |
|  | Attention mechanism | 88.58 | 86.89 | 87.51 | 88.41 | 88.37 | 98.36 |
|  | Transfer learning | 80.9 | 74.33 | 76.36 | 65.3 | 76.27 | 90.19 |

The Data in Figure 12

| Comparison of CED results between different datasets in simple backgrounds | | | | | | |
| --- | --- | --- | --- | --- | --- | --- |
| / | MME (%) | 1 | 2 | 3 | 4 | 5 |
| Test Images (%) | CelebA Dataset | 1.27 | 63.18 | 88.42 | 95.18 | 98.37 |
|  | MTFL Dataset | 1.35 | 59.31 | 88.21 | 94.78 | 97.45 |
|  | LFW Dataset | 1.42 | 62.19 | 88.19 | 95.11 | 98.18 |
|  | CNBC Dataset | 1.21 | 63.07 | 88.12 | 94.92 | 97.05 |
| Comparison of CED results between different datasets in complex backgrounds | | | | | | |
| / | MME (%) | 1 | 2 | 3 | 4 | 5 |
| Test Images (%) | CelebA Dataset | 0.15 | 18.16 | 57.34 | 84.67 | 95.57 |
|  | MTFL Dataset | 0.04 | 17.22 | 57.12 | 84.25 | 95.14 |
|  | LFW Dataset | 4.37 | 41.09 | 67.29 | 91.01 | 96.17 |
|  | CNBC Dataset | 3.11 | 40.88 | 67.02 | 90.83 | 96.01 |

The Data in Figure 13

| The accuracy of facial expression recognition using different facial recognition techniques for overall sampling | | | | | | | | |
| --- | --- | --- | --- | --- | --- | --- | --- | --- |
|  | Different expressions | Happy | Sad | Angry | Amazed | Neutral | Detest | Fear |
| Recognition Rate | Deep learning | 0.61 | 0.46 | 0.77 | 0.62 | 0.55 | 0.71 | 0.53 |
|  | Attention mechanism | 0.72 | 0.53 | 0.86 | 0.72 | 0.64 | 0.82 | 0.64 |
| The accuracy of facial expression recognition based on local sampling using different facial recognition techniques | | | | | | | | |
|  | Different expressions | Happy | Sad | Angry | Amazed | Neutral | Detest | Fear |
| Recognition Rate | Deep learning | 0.71 | 0.61 | 0.67 | 0.52 | 0.64 | 0.61 | 0.68 |
|  | Attention mechanism | 0.81 | 0.72 | 0.76 | 0.59 | 0.76 | 0.68 | 0.75 |
